# Cost effectiveness of a community based prevention and treatment of acute malnutrition programme in Mumbai slums, India

**DOI:** 10.1371/journal.pone.0205688

**Published:** 2018-11-09

**Authors:** S. Goudet, A. Jayaraman, S. Chanani, D. Osrin, B. Devleesschauwer, B. Bogin, N. Madise, P. Griffiths

**Affiliations:** 1 Loughborough University, School of Sport Exercise and Health Sciences, Loughborough, United Kingdom; 2 SNEHA, Mumbai, India; 3 UCL Institute for Global Health, London, United Kingdom; 4 Department of Epidemiology and Public Health, Sciensano, Brussels, Belgium; 5 Department of Veterinary Public Health and Food Safety, Faculty of Veterinary Medicine, Ghent University, Merelbeke, Belgium; 6 African Institute for Development Policy, Lilongwe, Malawi; ESIC Medical College & PGIMSR, INDIA

## Abstract

Children in slums are at high risk of acute malnutrition and death. Cost-effectiveness of community-based management of severe acute malnutrition programmes has been demonstrated previously, but there is limited evidence in the context of urban slums where programme cost structure is likely to vary tremendously.

This study assessed the cost-utility of adding a community based prevention and treatment for acute malnutrition intervention to Government of India Integrated Child Development Services (ICDS) standard care for children in Mumbai slums. The intervention is delivered by community health workers in collaboration with ICDS *Anganwadi* community health workers. The analysis used a decision tree model to compare the costs and effects of the two options: standard ICDS services with the intervention and prevention versus standard ICDS services alone. The model used outcome and cost data from the Society for Nutrition, Education & Health Action’s Child Health and Nutrition programme in Mumbai slums, which delivered services to 12,362 children over one year from 2013 to 2014. An activity-based cost model was used, with calculated costs based on programme financial records and key informant interviews. Cost data were coupled with programme effectiveness data to estimate disability adjusted life years (DALYs) averted.

The community based prevention and treatment programme averted 15,016 DALYs (95% Uncertainty Interval [UI]: 12,246–17,843) at an estimated cost of $23 per DALY averted (95%UI:19–28) and was thus highly cost-effective. This study shows that ICDS *Anganwadi* community health workers can work efficiently with community health workers to increase the prevention and treatment coverage in slums in India and can lead to policy recommendations at the state, and potentially the national level, to promote such programmes in Indian slums as a cost-effective approach to tackling moderate and severe acute malnutrition.

## Introduction

Children in slums are at high risk of acute malnutrition and its associated long-term negative consequences for growth and intellectual development. In Mumbai, India, forty one percent of the population (9 million) live in slums in which unhealthy conditions such as lack of access to safe water and sanitation, high density of population, and poor infrastructure pose serious health risks [1]. The Integrated Child Development Services (ICDS) is a welfare scheme launched in 1974 by the Government of India to tackle malnutrition and health problems in children below 6 years of age and their mothers. The programme offers health, nutrition and hygiene education to mothers, supplementary feeding for children and pregnant and breastfeeding mothers, growth monitoring and promotion, and links to primary healthcare services. The aim of this paper is to examine the cost effectiveness of the ‘*Aahar* acute malnutrition programme’, supported by the Society for Nutrition, Education and Health Action (SNEHA), to complement the ICDS scheme by providing community based prevention and treatment of acute malnutrition in children under 3 years old. The *Aahar* acute malnutrition programme approach is based on early screening of acute malnutrition through identification of all pregnant women and children younger than 3 years, growth monitoring, home-based counseling on feeding and care practices, referrals to public health-care facilities, treatment of malnourished children with provision of antibiotics and Medical Nutrition Therapy (MNT) (MNT is a local term for Ready to Use Therapeutic Food or RUTF). The *Aahar* acute malnutrition programme has demonstrated significant impact on wasting reduction; children in *Aahar* intervention areas were significantly less likely to be malnourished (adjusted odds ratio, 0.81; confidence interval, 0.67 to 0.99) [2] and improvement in exclusive breastfeeding practices [3].

The Community Management of Acute Malnutrition (CMAM) approach has typically four primary components: an outpatient therapeutic programme (OTP) for children with severe acute malnutrition (SAM) without complications, a stabilisation centre (SC) for short-term inpatient care, community mobilization, and—when appropriate—a supplementary feeding programme for children with moderate acute malnutrition. The *Aahar* acute malnutrition programme differs from the CMAM approach as it does not have its own OTP and SC (both are located in the tertiary municipal hospital, Lokmanya Tilak Municipal General Hospital near the intervention areas). The other differences are that *Aahar* acute malnutrition programme provides a day care centre for children with Moderate Acute Malnutrition (MAM) and Severe Acute malnutrition (SAM), at which they receive cognitive stimulation and therapeutic feeding. The intervention does not use Mid-Upper Arm Circumference (MUAC) for screening. Children who are no longer SAM or MAM are not discharged from the programme, but continue to be monitored by SNEHA and ICDS community health workers. SNEHA community health workers continued tracking their growth status via the mHealth platform.

Cost effectiveness analysis (CEA) studies of CMAM interventions or community based interventions have mostly been district-based in a mix of urban and rural settings [4–9]. Some of these interventions are pre-CMAM, and followed different standards. Cost effectiveness has been demonstrated in mixed urban-rural settings with delivery models through primary and secondary health care [4–6]. Other approaches work through health workers delivering services to the home [5, 7–9]. In a study of the cost-effectiveness of home-based treatment versus inpatient care in urban areas, Ashworth and Khanum [8], report that the average programme cost per child treated was US$29 for home-based care. This study, was the most similar approach to our own, in that the CMAM programme was delivered by community health workers, and showed high cost-effectiveness, with a cost per DALY averted of US$26 versus an inpatient care cost of US$1344. Costs to parents were lower for CMAM than for inpatient care. In Bachmann’s (2010) [10] systematic review of CMAM cost-effectiveness, the costs of outpatient community-based treatment of SAM ranged from US$46 to US$453 per child admitted. The costs of programmes with outpatient care and initial in-patient stabilization ranged from US$129 to US$201 per child admitted, and mortality proportion ranged from 1.2 to 9.2% [10]. Recent studies conducted after 2010 provide an average cost per DALY averted from US$26 to US$53 in Bangladesh, Malawi and Zambia.

We assessed the cost-effectiveness of a community-based programme for prevention and treatment of acute malnutrition delivered by non-government SNEHA community health workers. The aim of our study was to compare the cost-effectiveness of complementing the ICDS programme with the *Aahar* acute malnutrition programme to only ICDS standard care. The ICDS standard care scenario was estimated based on study data where *Aahar* acute malnutrition programme was not present [11, 12]. The cost-effectiveness of CMAM programmes, or prevention and treatment of acute malnutrition community based approaches with similar aims, has been demonstrated previously, but there are few data in the specific context of urban slums where programme cost structure is likely to be markedly different from rural areas. There is also a lack of information from the Indian context in which 65 million of the world’s urban slum populations reside [1].

## Methods

### Setting

SNEHA is a secular, Mumbai-based non-profit organization addressing four major areas of public health in urban slums: child health and nutrition, maternal and newborn health, sexual and reproductive health, and prevention of violence against women and children. The *Aahar* acute malnutrition programme is located in Dharavi, one of the largest urban slums in Asia with an estimated population of 700,000 to over 1 million [13]. Under-five mortality rates in slum areas are estimated at 27 per 1000 live births [14]. The prevalence of severe acute malnutrition (measured by Weight-For-Height <-3SD) is high: 4% of infants under two years old (0–24 months old, 95% CI: 2.7–6.0) suffer SAM [14, 15]. In a subsequent survey, 20% of infants under two years old were wasted (weight-for-height/length z <-2, 95% CI: 17.7, 22.2), 4.6% severely wasted (weight-for-height/length z <-3, 95%CI: 3.5, 5.6), and 18.8% were stunted (height-for-age z score <-2, 95%CI: 16.7, 20.9) [12] (MUAC and oedema were not used).

### Intervention characteristics

The CEA focused on one year during which the intervention was fully operational in five sectors of Dharavi. The project aimed to prevent and treat acute malnutrition in infants aged 0–3 years (more information on the project has been published in [2, 3]). Specifically, the *Aahar* acute malnutrition programme team identified six key result areas to reach these objectives: (1) prevent and treat acute malnutrition in infants under 3 years old; (2) increase in optimal breastfeeding practices among lactating mothers; (3) improve complementary feeding practices in infants 6 months to 3 years of age; (4) reduce infections and increase referrals and treatment of illnesses; (5) completion of immunizations; and (6) improve coverage of services provided by the ICDS. The programme monitoring system, using real time data collected via the mHealth platform, was based on these objectives and monthly reports against indicators were generated to track progress, identify gaps, and build adaptive strategies for optimal program performance. The indicators included were: 1) the total cumulative number of infants under three and pregnant women screened into the program, 2) the total number of pregnant women, SAM, MAM and Normal infants currently in the program, 3) the total number of infants who had left the program and reasons for leaving, 4) the population coverage of growth monitoring, 4) the number of home-based counseling visits to SAM infants, MAM infants, infants under 6 months, and pregnant women, 5) the number of SAM infants in OTP, 6) the number of SAM infants regularly consuming MNT. The mHealth platform used CommCare, a mobile application developed by Dimagi, USA [16].

The *Aahar* acute malnutrition programme was implemented in partnership with ICDS where 30 *Anganwadis (*one local ICDS child care centre per population of 1000*)* covered a total population of 30,000. ICDS standard activities in the communities included growth monitoring (to track weight-for-age), food distribution (take-home-rations), breastfeeding advice, complementary food advice and referral for immunization. In collaboration with the *Anganwadis* and government health workers, the programme was delivered by *Aahar* acute malnutrition programme community health workers who facilitated participation of caregivers and their infants younger than 3 years. Their responsibilities included facilitating growth monitoring of weight-for-height with ICDS, counselling, referrals, and support access to treatment. They received intensive training and were monitored and supervised closely by field supervisors based on real time performance tracking.

The programme had two components: treatment and prevention of acute malnutrition.

#### SAM and MAM treatment

The admission criteria for entering the SAM and MAM treatment protocol were: 1) anthropometric measurement: to be screened as MAM or SAM based on Weight-For-Height (WFH) (respectively <-2 SD and <-3SD), 2) age: be less than 3 years old and 3) location: living in the intervention area. The treatment component included provision of locally made Ready to Use Therapeutic Food—called Medical Nutrition Therapy (MNT) in this setting for SAM infants only, referrals to government health facilities for medical screening and immunization for MAM and SAM infants, growth monitoring and home-based counselling for MAM and SAM infants, and monitoring in daycare centres for MAM and SAM infants. ‘MNT cup’ is a locally produced Ready to Use Therapeutic Food (RUTF) made of peanut, soybean oil, skimmed milk powder, sugar, micronutrient premix and emulsifier produced by Nutrition Research and Rehabilitation Centre (NRRC). *Aahar*’s MNT provision characteristics: 8 weeks per child, once to twice daily, only for SAM children over 6 months who had medical screening and appetite test. The micronutrient content follows WHO recommendations and uses a micronutrient premix^18^. The nutritional composition per cup is as follow: energy 540 (kcal), proteins (g) 16, lipids (g) 34. The nutritional composition per 100g equivalent Plumpy’Nut (RUTF formule F100): energy (kcal) 543, proteins (g) 14, lipids (g) 36.

Daycare centres were physical places in the community, staffed by a teacher and helper to promote nutritional treatment for SAM/MAM infants. The treatment protocol included the following steps. Infants identified with SAM were referred to either the Nutritional Rehabilitation and Research Centre (NRRC) which is the equivalent of a stabilisation centre (as in CMAM) or to community-based health camps organised by SNEHA (equivalent of an OTP), their anthropometrics were recorded, an appetite test was administered, and a doctor examined the infants for medical complications. Infants with medical complications were referred to appropriate public health facilities. Infants with uncomplicated SAM were followed up for weight gain at home. Infants with SAM or MAM aged 15–36 m were optionally enrolled at SNEHA day care centres where they received cognitive stimulation and fortified snacks (porridge). In the home-based approach, infants with uncomplicated SAM were prescribed MNT by a doctor, based on the RUTF WHO reference chart, for a period of 8 weeks. If the child was unable to achieve the target weight in the first two weeks, further investigations were carried out for weight stagnation. If the child had not reached the target weight by the end of the intervention period, she was reassessed clinically. Drop-outs and irregular consumption were checked regularly and, if a mother or caretaker refused to go to the NRRC, attend a health camp, or give MNT to the child, the child was retained in the programme, but with counselling and weight monitoring only. If a child defaulted from the MNT 8-week protocol and consumed MNT irregularly, the mother or primary caregiver was counselled again on the importance of regular consumption. Issues were identified during programme implementation related to MNT consumption adherence due to poor taste and high density. If MNT was not consumed for a week or a few days, the number of days missed were added to the treatment duration (up to a maximum of one additional month). MNT was discontinued until recovery if the child had diarrhoea and the doctor was informed. Infants identified with SAM or MAM were referred to one of five municipal health posts in the area for immunization. Monthly health camps were organized in community spaces or day care centres by a medical doctor trained in SAM management. Infants with SAM were mobilized for health check-ups which included anthropometric measurement, diagnosis of illnesses, and review of diet patterns. Weekly follow-up of infants with severe and moderate acute malnutrition at their home was conducted to monitor nutritional status and provide nutrition counselling and health education for mothers. These weekly sessions were not always possible due to mother’s or caretaker’s lack of time or availability or willingness to participate. In some instances, the child was only weighed as the mother or caretaker refused home counselling; in other instances, the mother or caretaker refused both growth monitoring and home counselling.

The programme did not include discharge criteria and ‘cured’ infants remained in the programme and were followed in the prevention component based on real time data monitoring. Cured infants were: 1) SAM infants who improved to MAM or normal (Weight-for-Height z >-3 SD (WHO 2006 growth references) for at least 1 month over the 1 year period extended by the 3 following months as change in nutritional status may be delayed); 2) MAM infants who improved to normal (Weight-for-Height z >-2 SD for at least 1 month within 12 months extended by the 3 following months as change in nutritional status may be delayed).

#### Prevention component

Prevention of acute malnutrition included home-based counselling for pregnant women, monthly home-based counselling for caretakers of infants below six months of age for promotion of appropriate feeding practices, monthly growth monitoring for all infants aged 0–3 years, and community awareness and capacity building of *Anganwadi* workers. Infants under six months also had their immunizations monitored and referred to municipal health posts for required immunizations. During the monthly screening, infants’ growths were monitored using individual growth cards. Community activities, often in conjunction with government and international campaigns (e.g. breastfeeding week), were organised to raise awareness.

Training and capacity building of community health workers and ICDS staff focused on maternal and child health and nutrition issues to build the capacity of frontline staff and to develop knowledge about malnutrition and infant and young child feeding practice guidelines. Refresher training was conducted for deeper understanding and reinforcement of the key messages that needed to be delivered in the community. Additionally, community health workers benefited from additional training compared to the ICDS staff and were closely supervised and monitored by field supervisors. Supportive supervision employed performance tracking based on monthly target achievement (e.g. number of infants visited, number of infants weighed, number of pregnant women enrolled and visited, etc.).

### Decision tree model

**Fig 1:**
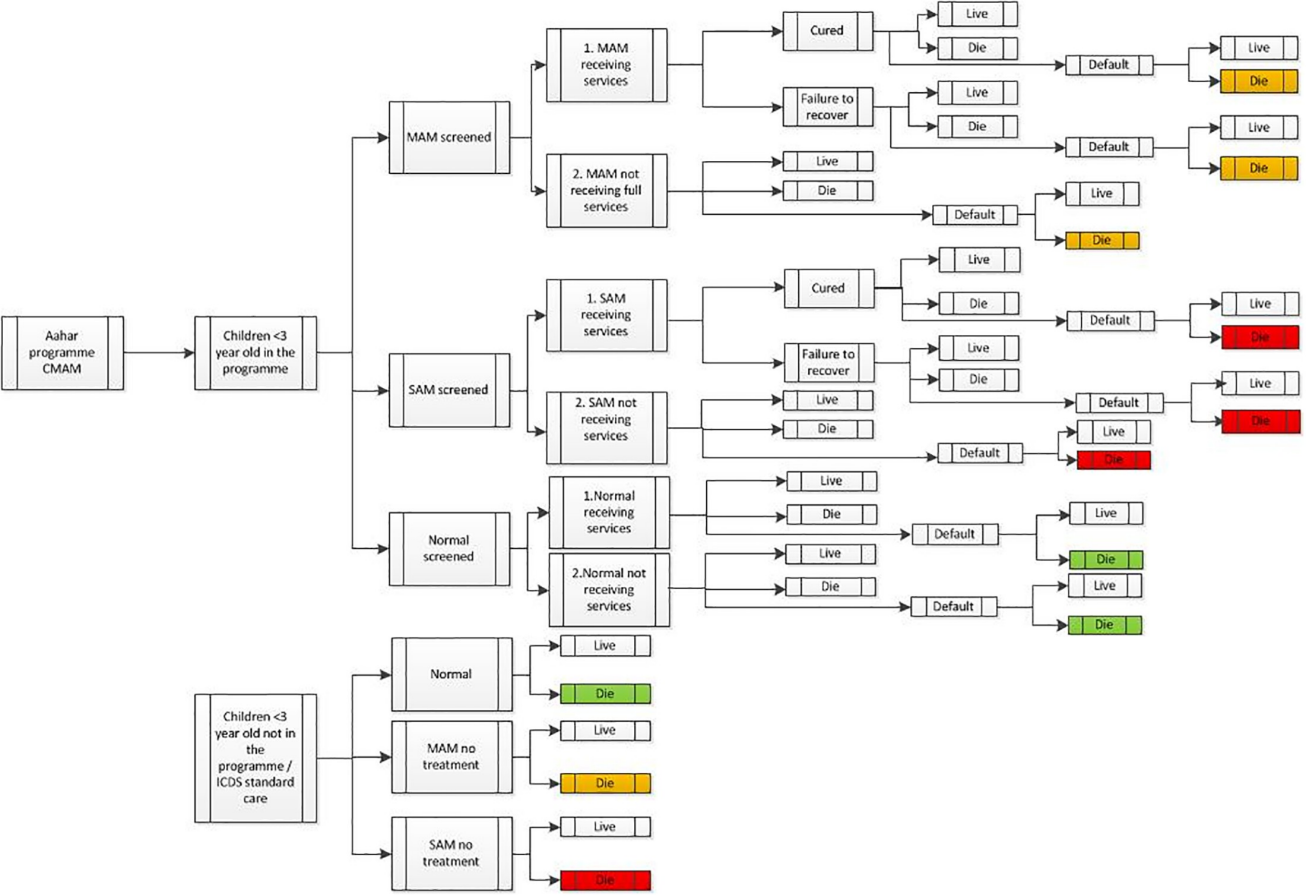
Decision tree CMAM and prevention for *Aahar* acute malnutrition programme. **Cured, SAM:** Improved to MAM or normal (Weight-for-Height z >-3 SD (WHO 2006 growth references) for at least 1 month over the 1 year period extended by the 3 following months as change in nutritional status may be delayed) **Cured, MAM**: Improved to normal (Weight-for-Height z >-2 SD for at least 1 month within 12 months extended by the 3 following months as change in nutritional status may be delayed) **Failure to recover, SAM**: Remained SAM. **Failure to recover, MAM**: Moved from MAM to SAM, or remained MAM over the 1 year period. **Failure to recover, Normal**: Moved from normal to MAM or SAM over the 1 year period. **Default, SAM, MAM or Normal**: Turned 3 years old, migrated, or was screened incorrectly. **Relapse, SAM**: Recovered but relapsed to SAM over the 1 year period. **Live, Children in the programme** (SAM, MAM or normal): Based on programme data **Live, Children not in the programme** (SAM no treatment, MAM no treatment or normal): Based on data from survey data.

A decision tree model (Fig 1) described the pathways along which a child might proceed if admitted to the treatment and prevention programme or if benefitting only from ICDS standard care.

Infants exited the programme in one of four ways: cured, death, default, or failure to recover. Infants of normal nutritional status benefitted from the prevention component. Infants with SAM and MAM who were not part of the programme and received standard ICDS services only were assumed to have the same mortality rates as untreated infants. The normal treatment path for MAM and SAM leading to ‘cured’ was “screened, receiving services as described in the treatment section” (1. in decision tree MAM / SAM ‘receiving services’). In some cases, MAM and SAM infants followed other paths that did not lead to ‘cured’ due to refusal of home visits and MNT consumption (2. in decision tree ‘not receiving full services’). The ‘normal’ infants received services for prevention (1. Normal infants receiving services). Screened normal infants were measured (weight and height) monthly after the initial screening and caretakers were counselled on optimal feeding practices and followed while the child was <6 m and if her mother was breastfeeding. Nevertheless, some of the screened normal infants did not receive all prevention services (2. Normal infants not receiving services); they were not measured if they were unavailable at the time of measurement or if their caregivers refused to have them weighed. Some of these infants only benefited from the community events in the area such as breastfeeding promotion. Finally, infants less than 3 years old who were not part of the programme were assumed to benefit from ICDS standard care only and were normal, MAM receiving no treatment or SAM receiving no treatment.

### Analytical costing

The analysis aimed to value the total costs of community treatment of SAM and MAM and prevention of acute malnutrition. An activity-based cost (ABC) analysis was used to estimate the total costs by cost centre. We identified cost centres by grouping activities that were related and validated them with SNEHA staff (S1 Table). They included all costs incurred by SNEHA and the households of infants with SAM. The treatment and prevention programme was evaluated for one year from February 2013 to January 2014 when the programme was fully operational. Costing was done using programme yearly costs, key documents including budget and financial reports, and key informant interviews with SNEHA staff (financial, programme, administrative, monitoring). The yearly costs of the treatment and prevention programme were the basis for the cost centres, with time allocation used to split staff salaries and associated costs across cost centres. Time allocation grids were designed based on interviews with SNEHA staff (community organiser, programme coordinator, programme officers, trainers). Activities for each profile were identified based on the post terms of references and were amended as necessary by the staff during an interview. The time spent was as well estimated on a daily and monthly basis. Interviews with the staff managers were conducted to check activities and time estimate. Cost centres allocation was then done using an allocation grid designed in collaboration with the financial team.

We grouped supervision costs in one cost centre to allow for comparison with other studies using similar grouping. All costs were expressed in local currency, and converted from Indian Rupees to US dollars using the July 2014 exchange rate when the analysis was conducted (US$ = INR 60.14). We used US dollars in order to compare our findings with those of previous studies.

Baseline and endline survey costs were not included. We assumed a 2-year shelf life for fixed assets. One-time setup costs were allocated at 50% (the residuum allocated to the programme covering other areas). We were not able to value the cost of ICDS standard care and assumed zero cost in the costing. The estimated costs of the programme therefore represent the incremental cost of adding such a programme to standard ICDS activities.

### Outcome assumptions

Outcome data for infants in the treatment and prevention programme were collected by the mHealth platform (Table 1).

**Table 1:** Treatment and prevention outcomes for *Aahar* acute malnutrition programme programme for girls and boys (admission, cured, death, default and relapse rate).

	all		Girls		Boys	
Outcome	n	%	n	%	n	%
Outcomes of children where *Aahar* was implemented						
**Total admissions (prevention + treatment)**	**12362**		**6014**	**49%**	**6348**	**51%**
**Total prevention admissions**	**8980**	**100.0%**	**4420**	**100.0%**	**4560**	**100.0%**
*Normal children getting services from programme*	6330	70.5%	3111	70.4%	3219	70.6%
*Normal children*: *death*	17	0.2%	10	0.2%	7	0.2%
*Normal children*: *default*	2633	29.3%	1299	29.4%	1334	29.3%
**Total treatment admissions**	**3382**	**100.0%**	**1594**	**100.0%**	**1788**	**100.0%**
*Children with SAM*	746	22.1%	332	20.8%	414	23.2%
*Children with MAM*	2636	na	1262	79.2%	1374	76.8%
*Children cured*	1888	55.8%	910	57.1%	978	54.7%
- Children with SAM cured	448	60.1%	206	62.0%	242	58.5%
- *Children with SAM cured but relapsed (included in the above)*	75	10.1%				
- *Children with MAM cured*	1440	54.6%	704	55.8%	736	53.6%
*Death*	8	0.2%	4	0.3%	4	0.2%
- *Children with SAM*	*5*	*0*.*1%*	*1*	*0*.*1%*	*4*	*0*.*2%*
- *Children with MAM*	*3*	*0*.*1%*	*3*	*0*.*2%*	*0*	*0*.*0% =*
*Default*	227	6.7%	105	6.6%	122	6.8%
*Treatment non-recovered*	1056	31.2%	476	29.9%	580	32.4%
*SAM or MAM not getting full services from programme (either no home visit and/or not weighted)*	203	6.0%	99	6.2%	104	5.8%
Death treatment + prevention	25	2.0%	14	2.3%	11	1.7%

Over the time of the study, 12,362 (49% female) infants were enrolled in treatment or prevention programmes, (8980 prevention, 3382 treatment). Of the infants admitted to the treatment programme, 1888 were cured (56%: 60% cured in infants with SAM and 55% cured in infants with MAM), 1056 (31%) failed to recover, 227 (8%) defaulted, 8 (0.2%) died and 203 (6%) did not receive full services (either receiving home visits but not being weighed, or not receiving home visits and not being weighed). Of the 1888 infants who were cured, 75 (18% of the SAM cured) relapsed into the SAM category during the study period of one year (Table 1). Here it is important to note that, unlike in a CMAM programme, the cured infants were not discharged and readmitted when screened for SAM again. These infants were still being monitored even after being considered as cured. Mortality ratios were 2% for the prevention and treatment programme, 1.9% for prevention only, 2.4% for treatment only (Table 1).

### DALY assumptions

DALYs are a measure of overall disease burden, expressing the number of healthy life years that are lost due to illness and death. Here, DALYs combined years of life lost (YLL) due to premature mortality related to SAM/MAM and years lived with acute malnutrition (YLD). For each node in the decision tree, either YLL or YLD were calculated using standard formulas for calculating DALYs based on YLD = number of prevalent cases * disability weighting * duration of the SAM episode and YLL = number of fatal cases * (residual life expectancy at mean age at death). Assumptions for calculation of YLL and YLD are described in Table 2. The probability and uncertainty of each outcome were calculated based upon programme data on mortality and recovery rates (Table 2) using similar methods to those of previous studies [5, 6]. We decided not to use discounting and age weighting in the base case scenario due to their controversial nature. Age weighting implies that the value of life depends on age while discount rate refers to the annual loss of value in percent. Nevertheless, to make the findings comparable to other studies [5, 6], we used the same parameters for age weight and discount rate as in these studies.

**Table 2:** Assumptions used to estimate Disability-Adjusted Life Years (DALYs) based on programme data and other evidence from previous CEA of nutrition studies.

	Units	Base case	Sources / assumptions	Sensitivity analysis: worst case	Sensitivity analysis: best case	Assumptions
**No intervention—ICDS standard care**						
Death rate untreated SAM (used for red nodes in decision tree)	%	7.6	Mortality rate of severely wasted under-fives average of 9 studies [17–26]	5.7	9.5	Worst and best estimate +/- 25%
Death rate untreated MAM (used for orange nodes in tree)	%	3.5	Mortality rate of moderately wasted under-fives average of 9 studies [17–26]	2.6	4.3	Worst and best estimate +/- 25%
Death rate normal weight children (use for green nodes in decision tree)	%	2.6	NFHS-3 India 2006 [11]	2.0	3.3	Worst and best estimate +/- 25%
Global Acute Malnutrition prevalence	%	20.2	SNEHA baseline data 2014 [12]	15.2	25.3	Worst and best estimate +/- 25%
SAM prevalence (used for purple node in decision tree)	%	4.4	SNEHA baseline data 2014 [12]	3.3	5.5	Worst and best estimate +/- 25%
MAM prevalence	%	15.8	SNEHA baseline data 2014 [12]	11.9	19.8	Worst and best estimate +/- 25%
**DALYs**						
Age weight		0		1		
Discount rate		0		0.03		
Disability Weight for death (YLL)		1	WHO 2004 [27]	Fixed		
Disability Weight for SAM and MAM (YLD) using different DW within the confidence interval for the various level of services		0.127 (0.081–0.183) SAM: 0.097–0.127 MAM: 0.073–0.097	GBD 2010 [28](severe wasting)	Fixed		
Age at start of episode	months (years)	16.2 (1.35)	Programme data	Fixed		
Age at death	months (years)	22.5 (1.87)	16.2m (age at SAM) + 6 month for untreated SAM episode = 22.2 months (as in [5])	Fixed		
Duration of SAM episode (YLD calculation)	months (years)	6	untreated cases (as in [5])	Fixed		
Life expectancy at an average age of death (males)	years	64.4	World Bank 2011 [29]	Fixed		
Life expectancy at an average age of death (females)	years	68	World Bank 2011 [29]	Fixed		

We used the mHealth programme data when possible. However, for age at death of infants with SAM, the small number of cases could not give us a reliable average. Thus, we estimated the age of death as the age at SAM + the duration of SAM episode based upon a previous study [5]. We used the expected mortality ratio in the population when infants exited the programme or received only ICDS standard care (2.6% in NFHS-3 [11]). For untreated, non-responder and defaulter SAM and MAM case fatality ratios (7.6% for SAM and 3.4% for MAM) were estimated based on Pelletier et al.’s study [23] averaging the death rate for SAM and MAM respectively from 9 studies [17–26]. The case fatality rate used for untreated SAM is lower than the ones used in other studies; 18.1% in Wilford [6], 20.7% in Puett et al. [5], 18% in Bachmann [4]. This can be explained by the fact that they were based on MUAC rather than WFH, with MUAC being a better predictor of mortality than WFH [30–32]. For normal infants who left the programme, death rate was based on infant mortality rate in slums (2.6% in NFHS-3 [11]). Following Bulti et al. [33], the number of SAM deaths averted was calculated as excess mortality * proportion of treated SAM cases cured by the programme * number of SAM cases treated by the programme.

We used the disability weighting for severe wasting established by the Global Burden of Disease 2010 project [28]. For the levels of services received for infants with SAM, we used the confidence interval to reflect lower and higher disability weight. We used the same disability weight for infants with MAM as there were no data in the literature, but also used the lower confidence limit to reflect lower disability weight.

### Data analysis and sensitivity/uncertainty analysis

Estimates of DALYs averted were calculated for prevention and treatment compared to standard ICDS care. The incremental cost-effectiveness ratio (ICER) for the treatment and prevention programme versus the ICDS standard care alone was calculated as the estimated cost per DALY averted as no ICDS related cost was included for both options. Based on the 2001 recommendation of the Commission on Macroeconomics and Health, the World Health Organization classifies interventions as ‘highly cost-effective’ for a given country if they avert a DALY for less than per capita gross national income (GNI) or gross domestic product (GDP), and cost-effective if they avert a DALY for less than 3 times GNI or GDP. In India, GNI per capita was 1570 US$ in 2013 [34].

Sensitivity analyses were conducted for base, worst and best case using assumptions in Table 2 and a +/-25% range. The worst case scenario was based on the 25% CI with all inputs varying together and presented the least favourable results.

Uncertainty analysis was undertaken to develop scenarios with Dirichlet probabilities assigned to each possible outcome. The Dirichlet distribution was the natural choice for modelling the uncertainty in the transition probabilities of the decision tree models, because each node could result in two or more mutually exclusive outcomes. The cost uncertainty analysis was conducted by assigning cost centre to outcomes in the decision tree. The average costs for the individual decision tree nodes were used.

Calculations of DALYs, costs, estimated cost per DALY averted, and related uncertainties were performed in R version 3.4.0 (R Core Team 2017) [35]. Uncertainty analyses were based on 10,000 iterations, and 95% uncertainty intervals (UI) were defined as the 2.5th and 97.5th percentiles of the resulting uncertainty distributions.

Potential risk of bias was addressed by taken into account uncertainty in the decision tree parameters using a Monte Carlo-based uncertainty analysis. Furthermore, we performed sensitivity analyses by altering the main mortality scenarios, and by performing alternative social weighting scenarios.

### Ethical approval

Ethical clearance was granted by the ethical committee of Bandra Holy Family Hospital and Medical Research centre in Mumbai. We complied with the Principles of Ethical Practice of Loughborough University.

## Results

Costing annual programme costs totalled US$ 335,126, with an estimated cost for treatment of US$187,887 (detailed costs per cost centre in S2 Table). The average costs per child for treatment and prevention were US$ 27.

### DALYs and cost effectiveness analysis

The number of DALYs averted by the programme was 15,016 (95%UI: 12,246–17,843) in the base scenario (9,479 worst case– 20,697 best case) (Table 3).

**Table 3:** Disability-Adjusted Life Years (DALYs), DALYs averted, and cost per DALY averted under different mortality scenarios (base, best and worst) for the community based treatment and prevention programme versus ICDS standard care only.

	Base (Confidence Interval)	Best (Confidence Interval)	Worst (Confidence Interval)
Aahar acute malnutrition programme	8,912 (7,494–10,425)	9,169 (7,732–10,673)	8,532 (7,155–10,039)
ICDS standard care	23,928 (21,610–26,406)	29,866 (27,308–32,501)	18,011 (15,982–20,151)
DALYs averted	15,016 (12,246–17,843)	20,697 (17,754–23,712)	9,479 (6,971–12,054)
Cost (USD) per DALY averted	23 (19–28)	16 (14–19)	36 (28–48)
SAM deaths averted	27 (11–43)	37 (20–55)	17 (3–32)
Cost (USD) per SAM death averted	12,630 (7,750–29,601)	9,713 (6,115–16,514)	26,724 (10,262–86,536)

This is the difference between the number of DALYs for the programme for base of 8,912 (95%UI: 7,494–10,425) and the infants less than 3 years old outside the programme (ICDS standard care imaginarily applied to the same cohort of individuals (i.e., the DALYs if those children would have undergone standard care), respectively 23,928 (95%UI: 21,610–26,406). The number of SAM deaths averted was 27 for base, (17 worst case– 37 best case). The estimated cost per SAM death averted was US$12,630 (US$27,724 worst case–US$9,713 best case). The main contributors to the averted DALYs were the normal infants who had been prevented from becoming malnourished (10,961 DALYs averted), the MAM infants (2,311 DALYs averted) and SAM infants (1,743 DALYs averted) (details in S3 Table). The fact that these are the main contributors are explained by the difference in malnutrition prevention and in assumed mortality for non-treatment between the 2 arms.

The ICER of implementing the treatment and prevention programme in addition to existing ICDS health services was, for base, estimated US$23 per DALY averted (95%UI:19–28) (US$36 worst case—US$16 best case). The scenario analysis using 3% time discount rate (S4 Table) shows that the number of DALYs averted was 6,658 (95%UI: 5,437–7,915) compared to 15,016 (95%UI: 12,246–17,843) with no time discounting. The ICER of implementing the treatment and prevention programme in addition to existing ICDS health services was, using a 3% time discount rate US$51 per DALY averted (95%UI: 43–62). When using 3% time discount rate and age weighting, the number of DALYs averted was 7,380 (95%UI: 6,393–9,307) and the ICER was US$43 per DALY averted (95%UI: 36–53).

## Discussion

The results suggest that a treatment and prevention programme could avert 15,016 DALYs at an estimated cost per DALY averted of US$ 23, at a total programme cost of US$335,126. We discuss effectiveness, cost-effectiveness in terms of cost per DALY averted, and the cost analysis of the intervention.

### Effectiveness (health outcomes of the programme)

Of the 3382 infants enrolled in the programme, 1888 were cured (treatment proportion of 56%, of which 76% had SAM and 24% had MAM). The infants who were not cured (44%) included 1056 (31%) who did not recover, 272 (8%) who defaulted, and 203 (6%) who did not receive full services (these infants did not benefit from growth monitoring). These performances are low in terms of cure and default rates according to SPHERE minimum standards and previous studies (Table 4). The SPHERE minimum standards are evidence-based and represent sector-wide consensus on best practice in humanitarian response.

**Table 4:** Performance indicators comparison of *Aahar* acute malnutrition programme with SPHERE and other CEA studies of nutrition interventions.

In %	Aahar acute malnutrition programme, India n = 3382	SPHERE standards	Gabouland et al. [9] Niger n = 340	Wilford et al. [6] Malawi n = 2896	Puett et al. [5] Bangladesh n = 724
Cure	**56 (based on WFH> = -2)**	>75 (based on 15% weight gain)	92.7 (based on 15% weight gain)	91.3 (based on 15% weight gain)	91.9 (based on 15% weight gain)
Death	**2.4**	<10	1.7	1.0	0.1
Failure to recover	**17.8**	<15	5.6	4.6	8.1
Transfer				3.1	
Not getting services	**6.0**				

Regarding the cure rate, comparison with previous studies should be made carefully as these used a criterion of 15% weight gain, while the *Aahar* acute malnutrition programme used WHZ ⩾ − 2. This is in line with the new WHO guidance issued in 2013 on acute malnutrition treatment. Compared to previous programmes using the old criteria of 15% weight gain, the use of WHZ ⩾− 2 as a recovery criterion explains the lower recovery rate. Aguayo et al. (2014) have shown that use of WHZ ⩾− 2 reduced recovery rates approximately twofold (17.5%) compared with the use of weight gain ⩾ 15%. The programme cure rate was in line with another large-scale CMAM programme in Bihar state, India that reported a 57% cure rate using the new WHO criterion [36]. The reasons for this low cure rate might be low food security at household level or the fact that infants may have been considered to have recovered too early. A suggested action might be to consider interventions at the household level to increase food security for vulnerable groups [37].

### Cost effectiveness and DALYs

The estimated cost of US$23 (95%UI: 19–28) per DALY averted suggests that the model of treatment and prevention is highly cost effective based on the WHO GDP per capita threshold (Commission on Macroeconomics and Health 2001) (in India, GNI per capita was 1570 US$ in 2013). This is the same as reported in CMAM or community based treatment programme studies in countries with similar GNI per capita (Bangladesh: US$1010, Zambia: US$1810 in 2013), US$ 27 (dollar value in 2015) per DALY averted for community treatment of SAM in Southern Bangladesh [5], and US$58 (dollar value in 2015) in Zambia [4]. The programme had similar cost effectiveness to other priority child health interventions and the high caseload of children admitted to the programme contributed to the high number of DALYs averted.

### Cost analysis

The overall programme cost was US$ 335,126 This is less than reported in Malawi with a similar number of admissions (US$ 470,703). The difference is explained by the high cost of RUTF in Malawi (US$ 148,519) versus (US$ 20,857) in *Aahar* acute malnutrition programme. In terms of cost centres, day care centre costs represented the greatest relative expense (14%), followed by supervision and follow-up (12% each). The importance of the day care centre to costs, and the fact that it was related only to infants with SAM and MAM should be taken into consideration in cost structure improvement: might the programme be as effective without day care centres? The estimated cost per death averted was US$13,977, much higher than reported in other studies (US$ 869 in [5], US$1760 in [4]) is mainly due to the lower cure rate.

In order to make comparisons, we grouped costs in comparable categories to other studies.

In this configuration, administration was the main cost item (39%), followed by technical support (29%), management (13%) and day care centres (8%). The high administrative share accrued because administrative costs included central office costs. The second greatest cost component was technical, at 29%, lower than in other programmes (39% in Zambia) [4]. Management costs represented 13% and were lower than in other studies: 53% in Southern Bangladesh [5], 34% in Zambia [4], and 51% in Malawi [6]. This suggests that the programme was not management-heavy. It relied on a light management structure spread across levels and on a close and successful partnership with ICDS and the Municipal Corporation of Greater Mumbai which facilitated implementation of the programme. RUTF contributed only 4% to total cost, versus 24–40% in other CMAM programmes [4–7, 38]. This low cost is explained by the fact that the RUTF was locally produced in Mumbai at low cost compared to other programmes that had to import or transport RUTF at a higher price (US$38 per child vs US$40—US$73 in [5, 7]) (1.5 cup on average per day *56 average number of days for treatment *30 INR cost per cup = 2520 INR or $38). Household costs (US$ 0.72 per child admitted) were low compared to previous studies (US$29 for domiciliary care per child in Dhaka) [8]. Since many of the other studies covered medicine and other costs for cases of SAM, this could be probably explained by the fact that household were not able to dedicate additional resources such as increasing food expenditure. The source of other cost savings could be the leverage and utilization of all public health facilities (use of local health centres and the outpatient therapeutic programme and the stabilisation centre at the tertiary Hospital).

### Limitations

Programme performance is routinely evaluated based upon six organisational indicators, but not upon SPHERE indicators. For this analysis, SPHERE standard indicators had to be recalculated to allow for comparison with previous studies.

The analysis used zero to value the cost of ICDS standard care due to unavailability of cost data of the programme. This is a limitation as it would have been more useful to reflect the full cost of the intervention (ICDS + Aahar acute malnutrition programme).

A potential bias in the cost calculation could be due to either the overestimation or underestimation of time spent by staff members on each activity.

### Recommendations

The strengths of the programme are cost-effective prevention and treatment of acute malnutrition in a large caseload of infants, leading to a high number of DALYs averted by complementing India’s national ICDS programme. Areas of improvement in the programme performance have been identified related to the low cure rate and further research should be done to investigate if the poor performance for cure rate is due to the service delivery (e.g. the non-standard definition of discharge as cured, the use of WHZ versus MUAC, the lack of adherence to RUTF) or to household level factors (e.g. food insecurity).

Various levels of service were provided within the community based treatment and prevention programme. This is not typically the case in CMAM programmes. Although weighing infants can give valuable information to the caretaker, the fact that some of these infants have SAM means that without appropriate treatment they will not recover and face a high risk of dying. In addition, the high number of infants who did not respond to the treatment provided means that SNEHA could explore how the programme could improve the quality of the services and standardize the level of services by better promotion of the intervention. This could be linked to improving the acceptability of locally made RUTF. Since this research, Action Against Hunger (ACF) has worked in collaboration with the production unit to improve its quality and bring it up to WHO standards. This should lead to improvement in organoleptic qualities and better acceptability by children.

The programme day care centres accepted only infants with MAM and SAM, but not all MAM and SAM were able to attend and to benefit from them and they constituted a dominant cost item. A day care centre is similar to a crèche: infants are looked after by trained staff with a remit to include cognitive stimulation. This type of day care centre incorporates aspects of supplementary feeding centres and a crèche structure that have been referred to in the literature as having positive impacts on infants’ nutritional status if well-operated (in Nepal [39]; in Brazil [40]). SNEHA had decided at the time of our assessment to phase out the day care component because of its cost.

### Policy implications

Our research provides more evidence on the cost structure at scale of a community based treatment and prevention programme. The evidence suggests that such a programme can cost-effectively treat and prevent SAM and MAM in infants. This knowledge can assist policy-makers in prioritizing interventions within national budget constraints. Financial and human resources necessary to ensure scale-up of this programme in these contexts must be mobilized and appropriately allocated. Decision makers at global, national and local levels can use the evidence presented here to promote community based prevention and treatment of acute malnutrition as an integrated component of primary health care packages and nutrition programmes in the large number of contexts broadly similar to Mumbai slums.

## Conclusion

Our study shows that *Anganwadi* workers can work with community health workers to increase community based treatment and prevention programme coverage in urban slums in India, providing evidence to increase coverage of such programme in cities as a cost-effective way of tackling SAM. The cost structure and the high number of infants being admitted mean that the programme is successful at low cost per DALY averted. In line with national and international guidelines, community based prevention and treatment of acute malnutrition programmes can be scaled up and lead to reduction in SAM prevalence in India by reinforcing the countrywide Integrated Child Development Services.

## Supporting information

S1 Table Cost centres and their descriptions for *Aahar* acute malnutrition programme. (DOCX)

S2 Table Detailed costs per cost centres and average costs per children in USD for the community based treatment and prevention programme. (DOCX)

S3 Table Mean Disability-Adjusted Life Years (DALYs), Years of Life Lost (YLL), Years of Life lived with Disability (YLD) for the community based treatment and prevention programme (Aahar acute malnutrition programme) versus ICDS standard care only (base scenario) disaggregated for SAM, MAM and normal. (DOCX)

S4 Table Disability-Adjusted Life Years (DALYs), DALYs averted, and cost per DALY averted under different social weighting scenarios comparing the treatment and prevention programme versus ICDS standard care. DALY[*K*;*r*] denotes the applied age weighting constant (*K*) and discount rate (*r*).
(DOCX)

S1 Database Database including all cases during the time period with data for anthropometric measurements. (XLSX)
