# Supplementary material for: Cost effectiveness of a community based prevention and treatment of acute malnutrition programme in Mumbai slums, India
Source: PLoS One. 2018 Nov 9;13(11):e0205688. doi: 10.1371/journal.pone.0205688 (PMC6226164; doi:10.1371/journal.pone.0205688)
Supplement: S1 Table — (DOCX) [file pone.0205688.s001.docx]

**S1 Table.** **Cost centres and their descriptions for *Aahar* acute malnutrition programme**

| **CC#** | **Cost centres** | **Description of the costs included** |
| --- | --- | --- |
| CC1 | Microplanning and household listing (done once at the beginning of the programme jointly with ICDS, to introduce the program objectives to the community, understand the intervention coverage area, and conduct a preliminary identification of children under three and pregnant women). | Community health workers' time spent conducting the microplanning and household listing in the community. |
| CC2 | Screening of all children under 3 y (monthly) | Community health workers' time spent conducting the screening. |
| CC3 | SAM anthropometry (once every 15 days) | Community health workers' time spent visiting households of children with SAM. |
| CC4 | Follow up visits for children < 6 m, pregnant or breastfeeding women, or counselling | Community health workers' time spent visiting households of children below 6 months and pregnant and breastfeeding women, and all printed materials and supplies used. |
| CC5 | SAM and MAM follow-up and counselling visits | Community health workers' time spent visiting households of children with SAM or MAM, and all printed materials and supplies used in case management of SAM or MAM. |
| CC6 | Therapeutic feeding | All curative care for SAM, including medicines and therapeutic foods (and its transportation and storage) for community management (daily or once in 1-2 days) and at day care centre, and equipment, medicines, food, bed and personnel costs at inpatient facility |
| CC7 | Daycare centre | All day care centre costs including staff, rental, supplies, medicines |
| CC8 | Training or capacity building of community health workers and ICDS staff | Salary, per diems of frontline workers and ICDS staff, transport and supplies. |
| CC9 | Survey (done quarterly for identification of new households and update of the house listing) | Community health workers' time spent conducting the screening. |
| CC10 | Community advocacy, events and campaigns | Community health workers' staff time, material and supplies |
| CC11 | Monitoring | Personnel and transportation costs incurred while monitoring and supervising frontline workers' during community case management of SAM. |
| CC12 | Supervision | Personnel and overhead costs for programme supervision at all levels of the programme. Proportion of time at monthly co-ordination meetings. |
| CC13 | mHealth (Commcare) | Cost of the mobile technology development and the smart phones used by the frontline workers. |
