# Supplementary material for: Cost effectiveness of a community based prevention and treatment of acute malnutrition programme in Mumbai slums, India
Source: PLoS One. 2018 Nov 9;13(11):e0205688. doi: 10.1371/journal.pone.0205688 (PMC6226164; doi:10.1371/journal.pone.0205688)
Supplement: S2 Table — (DOCX) [file pone.0205688.s002.docx]

**S2 Table.** **Detailed costs per cost centres and average costs per children in USD for the community based treatment and prevention programme**

| **CC** | **CC description** | **Sum of In Indian Rupees** | **Sum of In USD** | **Admin and overhead costs in USD** | **Total costs in USD** | **In %** | **Average costs for all children admitted in the programme in USD (normal, MAM, SAM)** |
| --- | --- | --- | --- | --- | --- | --- | --- |
| CC1 | Microplanning and house listing | 202,453.98 | 3,366.38 | 2,149.87 | 5,516.25 | 2% | 0.45 |
| CC2 | Screening of all children under 3 | 825,180.35 | 13,720.99 | 8,762.62 | 22,483.61 | 7% | 1.82 |
| CC3 | SAM and MAM anthropometry | 860,548.84 | 14,309.09 | 9,138.20 | 23,447.30 | 7% | 1.90 |
| CC4 | Follow up visits for children below 6 months | 922,246.29 | 15,334.99 | 9,793.37 | 25,128.36 | 7% | 2.03 |
| CC5 | SAM & MAM follow-up and counselling visits | 1,460,935.53 | 24,292.24 | 15,513.73 | 39,805.98 | 12% | 3.22 |
| CC6 | MNT Therapeutic Feeding | 765,474.97 | 12,728.22 | 8,128.61 | 20,856.83 | 6% | 1.69 |
| CC7 | Day care centre (DCC) | 1,683,754.30 | 27,997.24 | 17,879.86 | 45,877.10 | 14% | 3.71 |
| CC8 | Training /capacity building of frontline workers and ICDS staff | 736,722.86 | 12,250.13 | 7,823.29 | 20,073.42 | 6% | 1.62 |
| CC9 | Survey | 131,749.47 | 2,190.71 | 1,399.05 | 3,589.77 | 1% | 0.29 |
| CC10 | Community advocacy, events and campaigns | 800,164.91 | 13,305.04 | 8,496.98 | 21,802.02 | 7% | 1.76 |
| CC11 | Monitoring | 1,199,964.87 | 19,952.86 | 12,742.48 | 32,695.33 | 10% | 2.64 |
| CC12 | Supervision | 1,478,486.13 | 24,584.07 | 15,700.11 | 40,284.18 | 12% | 3.26 |
| CC13 | mHealth platform (Commcare) | 1,142,096.50 | 18,990.63 | 12,127.97 | 31,118.60 | 9% | 2.52 |
| CC14 | Household | 147,200.00 | 2,447.62 | - | 2,447.62 | 1% | 0.20 |
| Grand Total |  | 12,356,979.00 | 205,470.22 | 129,656.15 | 335,126.37 | 100% | 27.11 |
