# Supplementary material for: Cost effectiveness of a community based prevention and treatment of acute malnutrition programme in Mumbai slums, India
Source: PLoS One. 2018 Nov 9;13(11):e0205688. doi: 10.1371/journal.pone.0205688 (PMC6226164; doi:10.1371/journal.pone.0205688)
Supplement: S3 Table — (DOCX) [file pone.0205688.s003.docx]

**S3 Table. Mean Disability-Adjusted Life Years (DALYs), Years of Life Lost (YLL), Years of Life lived with Disability (YLD) for the community based treatment and prevention programme (Aahar acute malnutrition programme) versus ICDS standard care only (base scenario) disaggregated for SAM, MAM and Normal.**

| **Status** | **Components of DALY** | **Aahar acute malnutrition programme** | **ICDS standard care** | **Aahar acute malnutrition programme vs ICDS standard care** |
| --- | --- | --- | --- | --- |
| **Cause** | **Metric** | **Mean** | **Mean** | **Difference** |
| SAM | DALY | 1,317 | 3,060 | 1,743 |
| SAM | YLD | 36 | 35 | 0 |
| SAM | YLL | 1,281 | 3,024 | 1,743 |
| SAM | cases | 726 | 559 | -167 |
| SAM | deaths | 20 | 47 | 27 |
| MAM | DALY | 2,047 | 4,358 | 2,311 |
| MAM | YLD | 111 | 115 | 4 |
| MAM | YLL | 1,936 | 4,243 | 2,307 |
| MAM | cases | 2,606 | 1,813 | -793 |
| MAM | deaths | 30 | 66 | 36 |
| NRM | DALY | 5,548 | 16,510 | 10,961 |
| NRM | YLD | – | – | – |
| NRM | YLL | 5,548 | 16,510 | 10,961 |
| NRM | cases | 8,894 | 9,620 | 726 |
| NRM | deaths | 86 | 257 | 171 |
| **All** | **DALY** | 8,912 | 23,928 | 15,016 |
| **All** | **YLD** | 147 | 151 | 4 |
| **All** | **YLL** | 8,765 | 23,777 | 15,012 |
| **All** | **cases** | 12,226 | 11,992 | -234 |
| **All** | **deaths** | 136 | 370 | 234 |
