# Supplementary material for: Cost effectiveness of a community based prevention and treatment of acute malnutrition programme in Mumbai slums, India
Source: PLoS One. 2018 Nov 9;13(11):e0205688. doi: 10.1371/journal.pone.0205688 (PMC6226164; doi:10.1371/journal.pone.0205688)
Supplement: S4 Table — DALY[K;r] denotes the applied age weighting constant (K) and discount rate (r). (DOCX) [file pone.0205688.s004.docx]

**S4 Table. Disability-Adjusted Life Years (DALYs), DALYs averted, and cost per DALY averted under different social weighting scenarios comparing the treatment and prevention programme versus ICDS standard care.** DALY[*K*;*r*] denotes the applied age weighting constant (*K*) and discount rate (*r*).

|  | **DALY**  **(95%UI)** | **DALY**  **Social weighing used**  **(95%UI)** | **DALY**  **Social weighing and age discounting used**  **(95%UI)** |
| --- | --- | --- | --- |
| Aahar acute malnutrition programme | 8,912  (7,494–10,425) | 4,024  (3,397–4,691) | 4,602  (3,864–5,388) |
| ICDS standard care | 23,928  (21,610–26,406) | 10,682  (9,655–11,781) | 12,431  (11,222–13,725) |
| DALYs averted | 15,016  (12,246–17,843) | 6,658  (5,437–7,915) | 7,830  (6,393–9,307) |
| Cost (USD) per DALY averted | 23  (19–28) | 51  (43–62) | 43  (36–53) |
